# Predicting Significant Blood Pressure Reduction Through Ambulatory Blood Pressure Monitoring in Patients With Obstructive Sleep Apnea Treated With Continuous Positive Airway Pressure

**DOI:** 10.1111/crj.70167

**Published:** 2026-01-20

**Authors:** Yuanni Jiao, Hehe Zhang, Hao Wu, Xin Xi, Shuang Li, Jiang Xie

**Affiliations:** ^1^ Department of Respiratory and Critical Medicine Beijing Anzhen Hospital, Capital Medical University Beijing China; ^2^ Beijing Anzhen Hospital Centre for Sleep Medicine and Science Capital Medical University Beijing China

**Keywords:** ambulatory blood pressure monitoring, blood pressure, continuous positive airway pressure, obstructive sleep apnea

## Abstract

**Objective:**

Continuous positive airway pressure (CPAP) therapy for obstructive sleep apnea (OSA) results in a modest reduction in blood pressure. This study aimed to identify parameters from 24‐h ambulatory blood pressure monitoring (ABPM) that are predictive of treatment response.

**Methods:**

Treatment‐naïve patients with OSA were prospectively recruited from the Centre for Sleep Medicine and Science at Beijing Anzhen Hospital between July 2023 and April 2025. All participants underwent 24‐h ABPM assessments before and after 3‐month CPAP therapy. Correlations between the baseline ABPM data and post‐CPAP changes in blood pressure were analyzed. Multivariate analysis was used to determine whether specific baseline blood pressure cutoffs independently predicted a clinically significant reduction in blood pressure.

**Results:**

Good CPAP adherence (median usage: 6.1 h/night and 6.0 days/week; residual apnea–hypopnea index: 1.7 events/h) was achieved among 51 recruited patients (92.2% male, median age 40.5 years). After 3 months of CPAP treatment, significant reductions were observed in nearly all blood pressure measurements. Baseline 24‐h mean arterial pressure (MAP) was positively correlated with the reduction in all 24‐h blood pressure measures, all nighttime blood pressure measures, and daytime MAP. Compared with patients with 24‐h MAP < 96 mmHg, those with baseline 24‐h MAP ≥ 96 mmHg experienced relatively high absolute and relative reductions in all blood pressure measures.

**Conclusions:**

Baseline 24‐h MAP effectively predicts blood pressure reduction following CPAP therapy in patients with OSA, demonstrating the clinical value of an ABPM‐guided strategy for managing patients with comorbid OSA and hypertension.

Trial Registration: ChiCTR2300067728

## Graphical abstract

- CPAP therapy induces a modest reduction in blood pressure among patients with OSA.
- Patients with a baseline 24‐h MAP of ≥ 96 mmHg experience greater absolute and relative reductions in all blood pressure measures after CPAP treatment than those with a lower MAP.

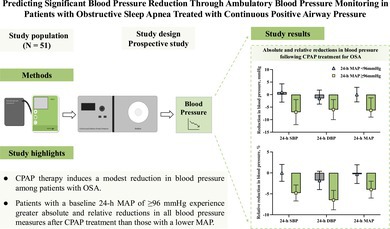

**Abbreviations**
ABPM: ambulatory blood pressure monitoring
AHI: apnea–hypopnea index
CPAP: continuous positive airway pressure
DBP: diastolic blood pressure
MAP: mean arterial pressure
OSA: obstructive sleep apnea
PTE: percent time elevation
SBP: systolic blood pressure

## 1 Introduction

Hypertension is a major global health burden, affecting over one billion adults aged 30–79 years. Despite its high prevalence, many individuals remain undiagnosed, and only a small proportion achieves adequate blood pressure control [1]. It is a major modifiable risk factor for cardiovascular disease and adverse outcomes. For example, among young adults, those with a 24‐h mean arterial pressure (MAP) exceeding 96 mmHg demonstrated a 2.14‐fold higher risk of cardiovascular events and a 2.53‐fold increase in the odds of cardiovascular mortality [2]. Targeted interventions that address the underlying causes of elevated blood pressure are crucial for slowing the progression of hypertension and preventing end‐organ damage.

Obstructive sleep apnea (OSA), a prevalent respiratory disorder marked by recurrent upper airway obstruction and intermittent hypoxemia, affects an estimated 936 million people worldwide, with a substantial proportion manifesting moderate‐to‐severe forms of the disorder [3]. OSA is recognized as a significant contributor to the development of hypertension. Continuous positive airway pressure (CPAP) remains the standard first‐line treatment for OSA, acting like an airway stent to maintain upper airway patency. Although CPAP is generally considered beneficial for blood pressure control, published data indicate only modest reductions in blood pressure [4, 5, 6, 7]. Previous analyses have demonstrated substantial heterogeneity in blood pressure response to CPAP therapy among patients with OSA, with greater reductions observed in those with uncontrolled hypertension at baseline, younger age, or more severe nocturnal oxygen desaturation [8]. Identifying the phenotypes of patients with comorbid OSA and hypertension who might experience a significant decrease in blood pressure post‐CPAP will provide insight on individualized therapeutic strategies, particularly for the non‐pharmaceutical treatment of early‐stage hypertension.

Current evidence indicates that elevated baseline blood pressure predicts a substantial reduction in blood pressure in response to CPAP therapy [7, 9]. However, existing studies include mixed populations of medicated and unmedicated patients, potentially confounding the results. The objective of this study was to assess treatment‐naïve patients with OSA, assessing the effect of CPAP on blood pressure and determining which baseline blood pressure parameters predict treatment response.

## 2 Methods

### 2.1 Study Population

In this observational cohort study, patients with suspected OSA who were referred to take a sleep test at Beijing Anzhen Hospital Centre for Sleep Medicine and Science between July 2023 and April 2025 were prospectively enrolled. The inclusion criteria were as follows: (1) 18–65 years of age; (2) not diagnosed with OSA before the screening; (3) not receiving CPAP treatment for any reason at the time; and (4) availability of staff and equipment. The exclusion criteria were as follows: (1) taking any antihypertensive agents at the time of recruitment; (2) secondary hypertension caused by renal artery stenosis, kidney disease, Cushing's syndrome, primary hyperaldosteronism, or pheochromocytoma; (3) acute coronary syndrome and high degree atrioventricular block; (4) rheumatic autoimmune diseases and malignant tumors; (5) aortitis, aortic dissection, or aneurysms; (6) diseases precluding an accurate measurement of blood pressure, such as atrial fibrillation and upper limb vascular diseases; (7) nasal obstruction unresponsive to nose sprays; and (8) rejected CPAP treatment.

**FIGURE 1:**
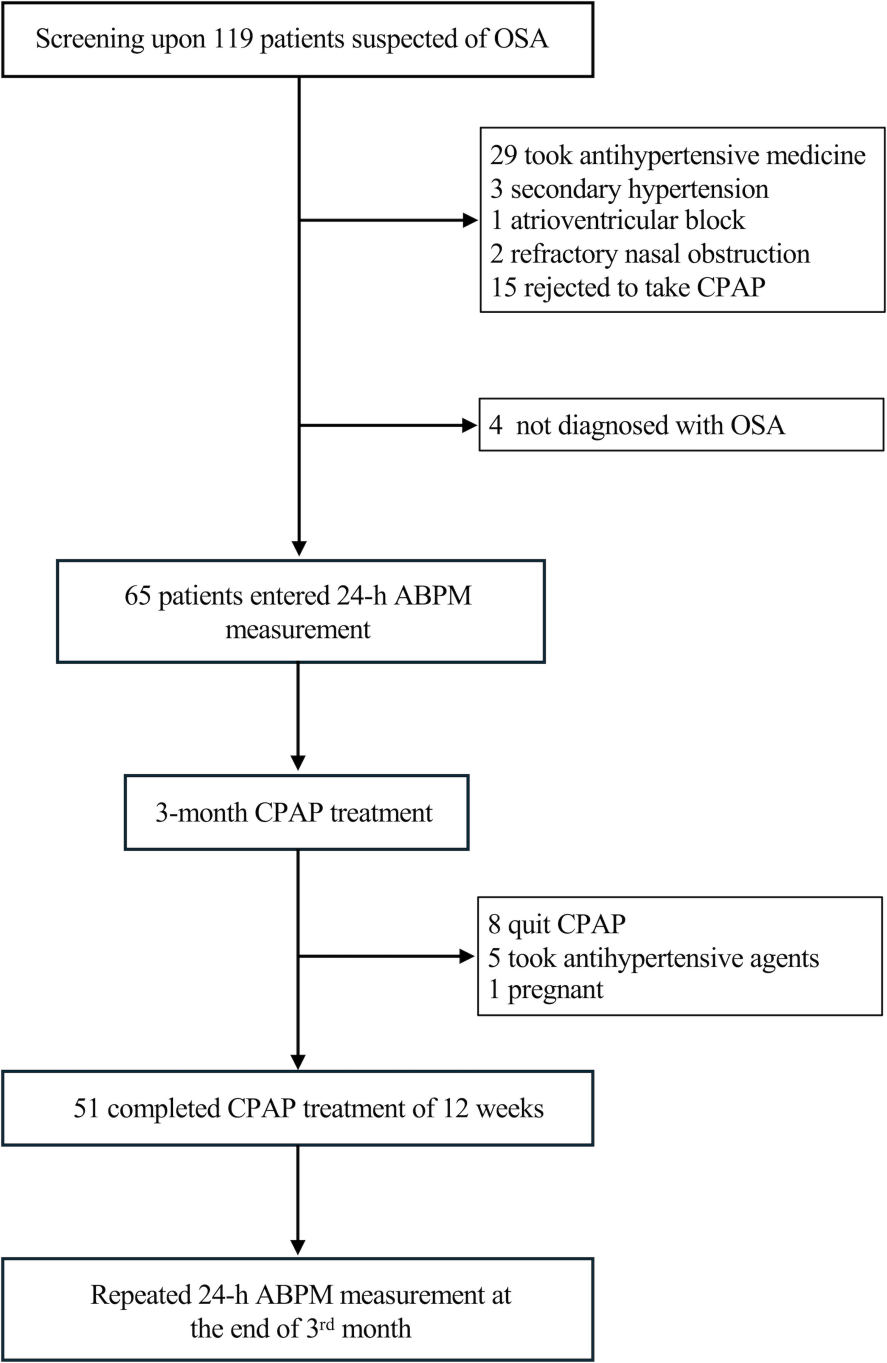
Flowchart of patient screening, treatment, and follow‐up. Abbreviations: ABPM = ambulatory blood pressure monitoring; CPAP = continuous positive airway pressure; OSA = obstructive sleep apnea.

Following the 119 screened patients, 54 were excluded (Figure 1), including four who did not meet the criteria for OSA. The remaining 65 patients were recruited for ABPM measurement and 3‐month CPAP treatment. Of these, 51 (78.5%) patients completed the 3‐month follow‐up, while 14 (21.5%) patients were excluded from the analysis (eight due to CPAP discontinuation, five who initiated antihypertensive medication, and one who was pregnant). This study was prospectively registered (ChiCTR2300067728) and approved by the Ethics Committee of Beijing Anzhen Hospital (IRB No. KS2022081). Participation was voluntary, and all subjects signed written informed consent forms.

### 2.2 Sleep Study

Fully attended overnight polysomnograms were performed using SOMNOtouch RESP (SOMNOmedics GmbH, Randersacker, Germany). Airflow parameters were assessed using nasal pressure sensors and oronasal thermal detection devices. Surface electrode arrays captured electrophysiological signals, including electroencephalography, electrooculography, and chin electromyography recordings. Respiratory movements of the chest and abdomen were measured using inductive plethysmography. Certified sleep specialists analyzed the collected data according to the standardized protocols of the American Academy of Sleep Medicine Manual for the Scoring of Sleep and Associated Events Version 3.0 [10]. Apneas were defined as ≥ 90% airflow reduction lasting ≥ 10 s, and hypopneas as ≥ 30% reduction in airflow lasting ≥ 10 s with ≥ 3% oxygen desaturation or arousal. The apnea–hypopnea index (AHI), representing the hourly average of combined breathing events, served as the diagnostic criterion for OSA (threshold: ≥ 5 events/h).

### 2.3 Office Blood Pressure Measurement

After completion of the overnight sleep test, trained medical staff measured the patients' office blood pressure the following morning using an ABPM device (ABPM‐05; Meditech Ltd., Budapest, Hungary). Before measurement, the patients were seated comfortably with back support and feet flat on the floor for at least 5 min and avoided caffeine, exercise, and smoking for at least 30 min. The arm was supported at the heart level with an appropriately sized cuff. Blood pressure was measured in the non‐dominant upper arm (most commonly on the left side). Two consecutive readings, taken 2 min apart, were averaged for analysis.

### 2.4 Ambulatory Blood Pressure Monitoring

To assess overall blood pressure levels and circadian rhythm, patients underwent 24‐h ABPM at baseline and again within 1 week following the 3‐month CPAP treatment. Participants were hospitalized for a 24‐h ABPM assessment to standardize pre‐ and post‐treatment conditions and minimize the influence of strenuous exercise on blood pressure readings. The device was fitted on the non‐dominant arm, and monitoring began at 3 pm and continued for 24 h. Blood pressure was measured every 15 min from 6 am to 10 pm (daytime) and every 30 min from 10 pm to 6 am (nighttime). The measurement was considered valid if ≥ 70% of the expected readings were obtained, namely ≥ 45 readings at daytime and ≥ 12 readings at nighttime.

The analysis focused on the 24‐h, daytime, and nighttime means of systolic blood pressure (SBP), diastolic blood pressure (DBP), and MAP. Hypertension was defined by 24‐h ABPM as a daytime SBP ≥ 135 mmHg or DBP ≥ 85 mmHg, a nighttime SBP ≥ 120 mmHg or DBP ≥ 70 mmHg, or a 24‐h SBP ≥ 130 mmHg or DBP ≥ 80 mmHg [11]. Percent time elevation (PTE) was defined as the percentage of total monitoring time during which blood pressure values exceeded the normal thresholds (SBP ≥ 135 mmHg or DBP ≥ 85 mmHg during daytime; SBP ≥ 120 mmHg or DBP ≥ 70 mmHg during nighttime) [12]. A reduction in blood pressure was defined as the follow‐up blood pressure minus the baseline value, and the relative reduction in blood pressure was calculated as the reduction in blood pressure divided by the corresponding baseline value. The nocturnal SBP decline rate was computed as (Daytime SBP − Nighttime SBP) / Daytime SBP × 100%. Blood pressure patterns were characterized as either dipping, defined as a ≥ 10% nocturnal SBP decline, or non‐dipping, defined by a decline of < 10% or any increase [13].

### 2.5 CPAP Treatment and Follow‐Up

Automatic pressure titration was conducted in‐lab for one or two nights to achieve an AHI of < 5 events/h using an auto‐CPAP device, and a 3‐month complimentary usage of the ResMed S9 AutoSet (Sydney, Australia) was provided to all participants. The participants were instructed to use the auto‐CPAP device for at least 4 h per night. The researchers emphasized the necessity of CPAP adherence and requested specialists from the departments of respiration and otolaryngology to address any issues affecting CPAP use during follow‐up. The CPAP usage data were downloaded during the first week of treatment and at the end of the follow‐up period. For patients with a residual AHI of ≥ 5 events/h, additional assessments and interventions were performed, including but not limited to checking for mask leaks and adjusting pressure.

### 2.6 Statistical Analysis

To determine the distribution of continuous data, the Shapiro–Wilk test was applied. Normally distributed variables were summarized as mean ± standard deviation and examined using Student's *t* test for between‐group differences and paired *t* tests for pre‐ and post‐CPAP comparisons. Variables with skewed distributions were described as median (interquartile range) and tested with the Mann–Whitney *U* method. Categorical outcomes were presented as counts (percentages) and analyzed using Pearson's chi‐square test. Spearman's correlation coefficient was employed to evaluate how baseline blood pressure values related to the magnitude of blood pressure reduction at the 3‐month follow‐up. To explore the blood pressure‐lowering effect of CPAP on patients across different baseline levels, patients were stratified according to baseline 24‐h MAP using a 96‐mmHg cutoff, based on a previously established prognostic threshold [2]. Data analysis and visualization were carried out using JMP 16 (SAS Institute, Cary, NC, United States). Results were interpreted with statistical significance defined as a two‐tailed *p* value of < 0.05.

## 3 Results

### 3.1 Patient Characteristics at Baseline

Baseline characteristics of the 51 study patients are summarized in Table 1. The majority of participants were male (*n* = 47, 92.2%), with a median age of 40.5 years. Hypertension, as determined by 24‐h ABPM, was present in 42 patients (82.4%). Patients with a baseline 24‐h MAP ≥ 96 mmHg exhibited higher values of body mass index (29.9 kg/m^2^ vs. 27.3 kg/m^2^, *p* = 0.007) than those with 24‐h MAP < 96 mmHg. Regarding polysomnographic parameters (Table S1), patients with MAP ≥ 96 mmHg had relatively high AHI (59.2 events/h vs. 42.6 events/h, *p* = 0.032) and oxygen desaturation index (57.1 events/h vs. 37.7 events/h, *p* = 0.010), relatively low minimum oxygen saturation (68.6% vs. 76.9%, *p* = 0.007), relatively frequent total arousal (51.9 events/h vs. 40.5 events/h, *p* = 0.029) and respiratory arousal (25.6 events/h vs. 10.9 events/h, *p* = 0.039), and a relatively high Epworth Sleepiness Scale score (13.4 vs. 10.0, *p* = 0.023).

**TABLE 1:** Baseline clinical characteristics of the study patients.

Variables	All patients (*n* = 51)	Patients with baseline 24‐h MAP < 96 mmHg (*n* = 20)	Patients with baseline 24‐h MAP ≥ 96 mmHg (*n* = 31)	*p*
Age, (years)	40.5 ± 8.1	42.4 ± 8.8	39.3 ± 7.5	0.204
Male, *n* (%)	47 (92.2)	17 (85.0)	30 (96.8)	0.287
Hypertension based on ABPM, *n* (%)	42 (82.4)	11 (55.0)	31 (100.0)	< 0.001
Diabetes/Impaired glucose tolerance/Impaired fasting glucose, *n* (%)	16 (31.4)	7 (35.0)	9 (29.0)	0.760
Urinary microalbumin, (mg/L)	17.3 (8.0, 57.1)	16.6 (8.7, 35.5)	24.1 (6.9, 61.0)	0.602
Current/Past smoker, *n* (%)	24 (47.1)	12 (60.0)	12 (38.7)	0.161
Body mass index, (kg/m^2^)	29.3 (27.3, 33.4)	27.3 (25.2, 29.3)	29.9 (28.1, 33.8)	0.007
Neck circumference, (cm)	41.9 ± 3.3	40.7 ± 3.8	42.7 ± 2.7	0.057
Abdominal circumference, (cm)	104.7 ± 12.2	100.6 ± 12.0	107.4 ± 11.7	0.052
Modified Mallampati classification				
Normal (I/II), *n* (%)	11 (21.6)	5 (45.5)	6 (54.6)	0.733
Abnormal (III/IV), *n* (%)	40 (78.4)	15 (75.0)	25 (80.7)	0.733
24‐h MAP, (mmHg)	99.0 ± 10.2	88.7 ± 6.2	105.7 ± 5.7	< 0.001
24‐h heart rate, (bpm)	79.2 ± 9.9	76.4 ± 9.7	81.0 ± 9.7	0.105

*Note:* Results are expressed as mean ± standard deviation or median (interquartile range), or *n* (%).

Abbreviations: ABPM = ambulatory blood pressure monitoring; MAP = mean arterial pressure.

### 3.2 Blood Pressure Response to CPAP Therapy

During the 3‐month follow‐up, patients demonstrated good CPAP adherence (median usage: 6.1 h/night and 6.0 days/week). The median and 95th percentile of CPAP therapeutic pressures were 9.0 ± 2.1 cmH_2_O and 11.5 ± 2.3 cmH_2_O, respectively, and a residual AHI of 1.7 events/h was achieved (Table S2).

**FIGURE 2:**
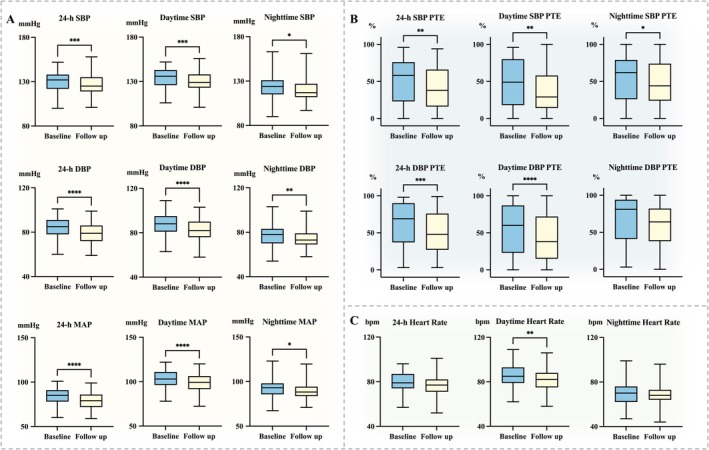
Changes in blood pressure, percent time elevation, and heart rate from baseline to 3‐month follow‐up. (A) SBP, DBP, and MAP (24‐h, daytime, and nighttime) were significantly reduced after treatment. (B) PTE was significantly reduced for SBP (24‐h, daytime, and nighttime) and DBP (24‐h and daytime). (C) Daytime heart rate decreased significantly. Data are presented as box‐and‐whisker plots, with baseline values presented on the left and 3‐month follow‐up values on the right. *p < 0.05; **p < 0.01; ***p < 0.001; ****p < 0.0001. Abbreviations: DBP = diastolic blood pressure; MAP = mean arterial pressure; PTE = percent time elevation; SBP = systolic blood pressure.

After 3 months of CPAP treatment, significant reductions from baseline were observed in nearly all blood pressure measures, including 24‐h, daytime, and nighttime SBP, DBP, and MAP (Figure 2A). PTE values were significantly reduced for both SBP (24‐h, daytime, and nighttime SBP) and DBP (24‐h and daytime DBP) (Figure 2B). In addition, the daytime heart rate decreased significantly, whereas the 24‐h and nighttime heart rates showed no significant changes (Figure 2C).

### 3.3 Factors Associated With CPAP‐Induced Blood Pressure Reduction

**FIGURE 3:**
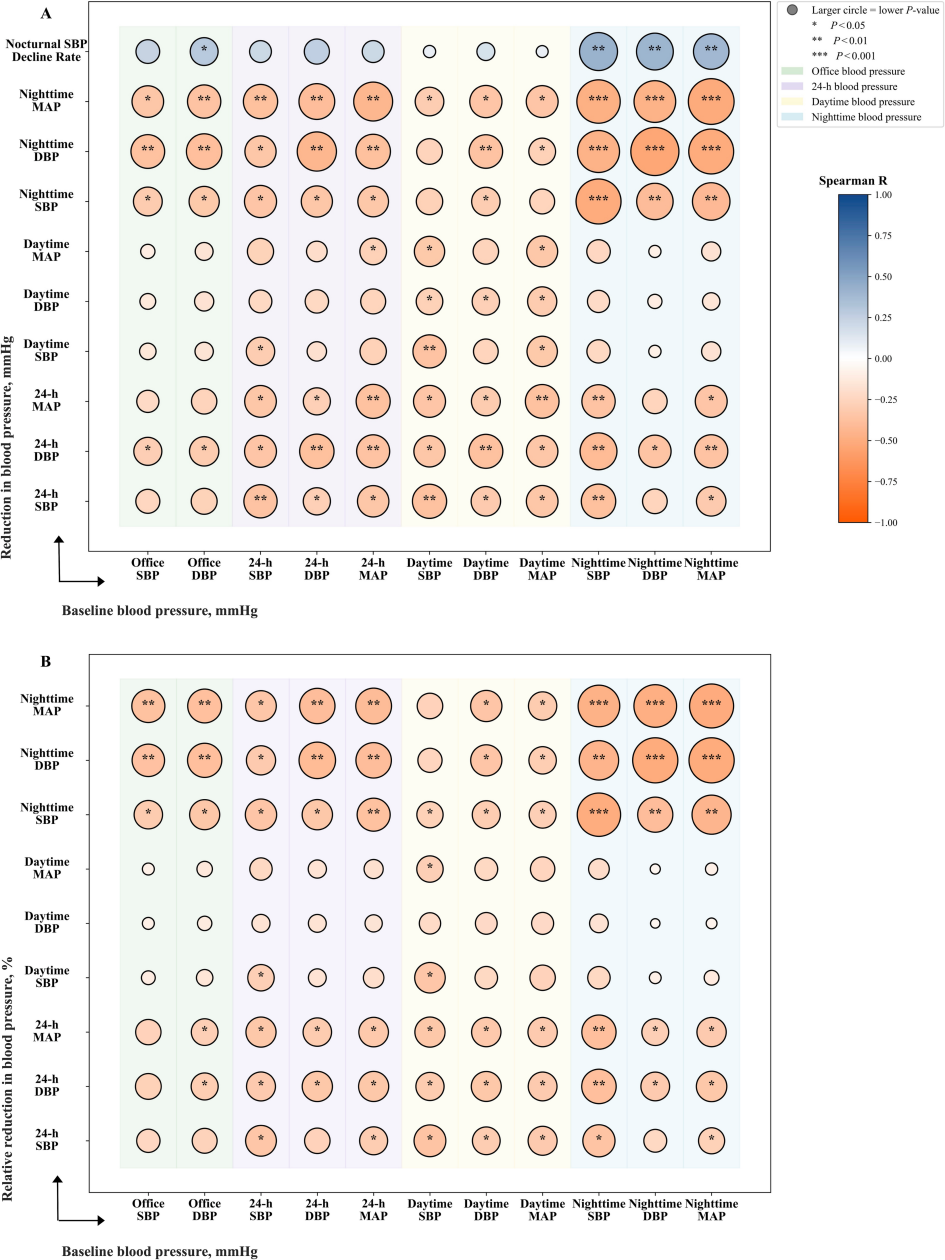
Correlations between baseline blood pressure and reduction in blood pressure at 3‐month follow‐up. (A) Correlation between baseline blood pressure and absolute reduction in blood pressure indices. (B) Correlation between baseline blood pressure and relative reduction in blood pressure indices. Bubble size indicates the significance level (larger circles denote smaller *P*), and color represents the Spearman correlation coefficient (red = positive, blue = negative). *p < 0.05; **p < 0.01; ***p < 0.001. Abbreviations: DBP = diastolic blood pressure; MAP = mean arterial pressure; SBP = systolic blood pressure.

Overall, baseline blood pressure measurements demonstrated mild‐to‐moderate correlations with the magnitude of blood pressure reduction observed at the 3‐month follow‐up (Figure 3A). Notably, the baseline 24‐h MAP was positively correlated with the reduction in all 24‐h blood pressure measures, all nighttime blood pressure measures, and daytime MAP. Meanwhile, all baseline nighttime blood pressure measurements were positively correlated with a reduction in all nighttime blood pressure readings and the nocturnal SBP decline rate. Similar correlations were consistently observed for relative reductions in blood pressure, as shown in Figure 3B, with higher baseline 24‐h MAP and nighttime blood pressure measures associated with greater relative reductions in blood pressure, indicating that patients with higher baseline blood pressure tended to achieve larger proportional improvements after 3 months of CPAP treatment.

Moreover, compared with patients with a baseline 24‐h MAP < 96 mmHg, those with a baseline 24‐h MAP ≥ 96 mmHg exhibited a significantly greater reduction in blood pressure after 3 months of CPAP treatment (Table 2). These differences were largely consistent across all blood pressure measurements, that is, 24‐h, daytime, and nighttime readings. In addition, patients with baseline 24‐h MAP ≥ 96 mmHg exhibited substantial reductions in 24‐h SBP and DBP PTE.

**TABLE 2:** Magnitude of blood pressure reduction in CPAP‐treated patients by baseline MAP.

Blood pressure measures	All patients (*n* = 51)	Patients with baseline 24‐h MAP < 96 mmHg (*n* = 20)	Patients with baseline 24‐h MAP ≥ 96 mmHg (*n* = 31)	*p*
24‐h SBP reduction, (mmHg)	−4.0 ± 7.0	−0.2 ± 5.1	−6.5 ± 7.1	< 0.001
24‐h DBP reduction, (mmHg)	−4.1 ± 5.4	−1.5 ± 3.6	−5.8 ± 5.7	0.002
24‐h MAP reduction, (mmHg)	−3.8 ± 6.0	−0.4 ± 4.4	−6.0 ± 5.9	< 0.001
Daytime SBP reduction, (mmHg)	−3.7 ± 7.5	−0.7 ± 5.9	−5.7 ± 7.9	0.013
Daytime DBP reduction, (mmHg)	−4.7 ± 5.5	−2.7 ± 4.5	−6.0 ± 5.8	0.027
Daytime MAP reduction, (mmHg)	−4.1 ± 6.5	−1.3 ± 5.9	−5.9 ± 6.2	0.012
Nighttime SBP reduction, (mmHg)	−2.0 (−9.0, 4.0)	2.5 (−4.8, 8.8)	−6.0 (−10.0, 1.0)	0.010
Nighttime DBP reduction, (mmHg)	−3.0 (−8.0, 2.0)	0.5 (−3.8, 5.8)	−5.0 (−8.0, −1.0)	0.006
Nighttime MAP reduction, (mmHg)	−2.3 (−7.0, 2.0)	1.2 (−2.8, 6.8)	−4.7 (−8.7, −1.3)	0.001
24‐h SBP relative reduction, (%)	−2.9 ± 5.4	0.0 ± 4.3	−4.8 ± 5.2	0.001
24‐h DBP relative reduction, (%)	−4.7 ± 6.1	−1.8 ± 4.7	−6.5 ± 6.3	0.004
24‐h MAP relative reduction, (%)	−3.6 ± 5.9	−0.3 ± 4.8	−5.8 ± 5.6	< 0.001
Daytime SBP relative reduction, (%)	−2.7 ± 5.5	−0.6 ± 4.8	−4.0 ± 5.5	0.023
Daytime DBP relative reduction, (%)	−5.1 ± 6.0	−3.3 ± 5.6	−6.3 ± 6.0	0.080
Daytime MAP relative reduction, (%)	−3.8 ± 6.1	−1.4 ± 6.3	−5.3 ± 5.6	0.028
Nighttime SBP relative reduction, (%)	−1.5 (−7.3, 3.6)	2.2 (−4.0, 8.3)	−5.0 (−7.6, 0.9)	0.009
Nighttime DBP relative reduction, (%)	−3.7 (−9.0, 2.5)	0.7 (−5.8, 9.6)	−6.4 (−9.8, −1.1)	0.007
Nighttime MAP relative reduction, (%)	−2.6 (−7.2, 2.3)	1.3 (−3.2, 9.9)	−4.8 (−8.5, −1.4)	0.002
24‐h SBP PTE reduction, (%)	−9.3 ± 19.4	0.2 ± 14.8	−15.4 ± 19.7	0.002
24‐h DBP PTE reduction, (%)	−7.0 (−24.0, 4.0)	4.5 (−13.8, 11.5)	−10.0 (−34.0, −1.0)	0.006

*Note:* Results are expressed as mean ± standard deviation or median (interquartile range). ^+^ indicates blood pressure increase from baseline; ^−^ indicates blood pressure decrease from baseline.

Abbreviations: DBP = diastolic blood pressure; MAP = mean arterial pressure; PTE = percent time elevation; SBP = systolic blood pressure.

Beyond achieving a greater absolute decrease in blood pressure, patients whose baseline 24‐h MAP was ≥ 96 mmHg also demonstrated a significantly larger relative reduction across all blood pressure measures than patients with an MAP < 96 mmHg (Table 2). Notably, in the patient group with MAP ≥ 96 mmHg, three patients (9.7%) achieved full normalization of blood pressure (24‐h < 130/80 mmHg with daytime < 135/85 mmHg and nighttime < 120/70 mmHg) during follow‐up.

Patients with CPAP usage ≥ 6.0 h (*n* = 29) and those with usage < 6.0 h (*n* = 22) demonstrated similar reductions in all measured blood pressure parameters. In contrast, patients (*n* = 33) with a non‐dipping nocturnal blood pressure pattern showed a greater reduction in nighttime SBP, DBP, and MAP than those (*n* = 18) with a dipping pattern (Table 3).

**TABLE 3:** Blood pressure reduction in CPAP‐treated patients by nocturnal blood pressure profile.

Blood pressure measures	Nocturnal blood pressure profile		*p*
	Dipping (*n* = 18)	Non‐dipping (*n* = 33)	
24‐h SBP reduction, (mmHg)	−4.3 ± 6.5	−3.8 ± 7.4	0.787
24‐h DBP reduction, (mmHg)	−3.7 ± 4.9	−4.4 ± 5.7	0.636
24‐h MAP reduction, (mmHg)	−3.8 ± 5.2	−3.8 ± 6.5	0.942
Daytime SBP reduction, (mmHg)	−6.3 ± 7.1	−2.4 ± 7.5	0.073
Daytime DBP reduction, (mmHg)	−4.0 (−13.0, −2.0)	−4.0 (−8.0, 0)	0.281
Daytime MAP reduction, (mmHg)	−4.2 (−12.8, −1.8)	−3.3 (−7.7, 0.7)	0.211
Nighttime SBP reduction, (mmHg)	1.6 ± 6.4	−6.2 ± 10.7	0.002
Nighttime DBP reduction, (mmHg)	0.3 ± 6.0	−5.3 ± 8.8	0.009
Nighttime MAP reduction, (mmHg)	−1.5 (−4.2, 6.3)	−3.3 (−8.8, 1.0)	0.027
24‐h SBP relative reduction, (%)	−3.2 ± 5.1	−2.8 ± 5.6	0.816
24‐h DBP relative reduction, (%)	−4.0 ± 5.6	−5.0 ± 6.4	0.567
24‐h MAP relative reduction, (%)	−3.7 ± 5.2	−3.6 ± 6.3	0.984
Daytime SBP relative reduction, (%)	−4.5 ± 5.1	−1.7 ± 5.5	0.081
Daytime DBP relative reduction, (%)	−6.4 ± 6.0	−4.5 ± 6.0	0.290
Daytime MAP relative reduction, (%)	−5.6 ± 5.4	−2.8 ± 6.4	0.116
Nighttime SBP relative reduction, (%)	1.6 ± 5.6	−4.6 ± 8.4	0.003
Nighttime DBP relative reduction, (%)	1.0 ± 8.4	−6.0 ± 10.5	0.012
Nighttime MAP relative reduction, (%)	−1.5 (−4.4, 9.3)	−3.7 (−8.6, 1.1)	0.008

*Note:* Results are expressed as mean ± standard deviation or median (interquartile range). ^+^ indicates blood pressure increase from baseline; ^−^ indicates blood pressure decrease from baseline.

Abbreviations: DBP = diastolic blood pressure; MAP = mean arterial pressure; SBP = systolic blood pressure.

## 4 Discussion

Our study revealed that patients with comparatively high baseline blood pressure exhibited a relatively pronounced blood pressure reduction following CPAP therapy. We propose that a cutoff value of 24‐h MAP ≥ 96 mmHg, a threshold previously linked to increased cardiovascular risk [2], effectively identifies patients with OSA who derive greater blood pressure‐lowering benefits from CPAP therapy. The findings support the role of ABPM in guiding therapeutic individualization for patients with comorbid hypertension and OSA.

Our study reaffirmed the high prevalence of undiagnosed hypertension among patients with OSA, as detected using ABPM. However, the observed blood pressure reduction with CPAP treatment was modest, consistent with previous findings [14, 15]. For instance, Gottlieb et al. reported reductions of 1.9, 2.8, and 2.4 mmHg in 24‐h SBP, DBP, and MAP, respectively [14]. While extended CPAP use has been recommended for hypertensive patients comorbid with OSA [16, 17], excellent adherence with minimal residual AHI yielded only modest reductions in daytime MAP (4.1 mmHg) and nighttime MAP (2.3 mmHg) in our cohort. Notably, some patients experienced an increase in blood pressure after CPAP. These findings highlight the need to identify specific patient subgroups that may gain significant blood pressure‐lowering benefits from CPAP, thereby enabling a more personalized treatment approach.

Correlation analysis confirmed a strong association between baseline blood pressure and the magnitude of post‐CPAP reduction in blood pressure. To provide a clinically useful predictor, we adopted a well‐established cardiovascular prognostic threshold (24‐h MAP ≥ 96 mmHg) [2]. Patients above this threshold demonstrated relatively high reductions in both absolute and relative blood pressure measures. Previous investigations have similarly demonstrated that the blood pressure–lowering effect of CPAP is most evident in individuals with elevated baseline values, in agreement with our findings [7, 9]. In contrast with the aforementioned cohorts, our study population consisted of relatively young Asian patients who were antihypertensive‐naïve and free of medication during the follow‐up period. We propose that the implementation of this ABPM‐derived threshold in clinical practice could facilitate earlier identification of high‐risk patients with comorbid OSA and newly diagnosed hypertension who are most likely to benefit from CPAP therapy. This approach may help curb disease progression before the onset of target organ damage.

Notably, our study demonstrated a significant nocturnal blood pressure reduction in patients with a non‐dipping pattern, reaffirming the value of CPAP therapy for this cohort [18]. This finding further supports the use of 24‐h ABPM in OSA, given its essential role in assessing both blood pressure levels and circadian patterns to identify candidates most likely to benefit from CPAP.

Our study revealed several clues regarding the mechanism of significant blood pressure reduction in the patient group with 24‐h MAP ≥ 96 mmHg. First, they tend to have greater obesity, more frequent respiratory events, and more severe desaturation, which are closely associated with metabolic dysregulation and may be partially reversible through CPAP therapy [19, 20]. Second, a higher total and respiratory‐related arousal index and excessive daytime sleepiness, both of which significantly contribute to blood pressure elevation in OSA [21, 22], were observed in this patient group. By mitigating OSA‐induced arousal, excessive daytime sleepiness, and sympathetic overactivity, CPAP likely mediates significant improvements in blood pressure in this subgroup of patients.

Although most patients with a 24‐h MAP ≥ 96 mmHg (*n* = 31) achieved significant blood pressure reduction, only three attained full normalization across all daytime, nighttime, and 24‐h blood pressure measures. This result suggests that while CPAP effectively lowers blood pressure in hypertensive patients with OSA, only a small subset may avoid antihypertensive medications, whereas the majority will require adjunct therapies, such as lifestyle modifications or pharmacotherapy. In contrast, while our findings revealed the limited antihypertensive effect of CPAP in patients with OSA and 24‐h MAP < 96 mmHg, they do not negate its therapeutic value in moderate‐to‐severe OSA. Treatment decisions should remain guided by the overall cardiovascular risk (e.g., hypoxic burden) and symptom manifestations (e.g., daytime sleepiness) rather than blood pressure alone.

From a cardiovascular prognosis perspective, identifying patients with MAP ≥ 96 mmHg is clinically meaningful. This group carries a relatively high inherent risk and stands to gain the most from CPAP‐induced reduction in blood pressure. Early intervention in this population may delay hypertension progression and reduce long‐term cardiovascular morbidity.

## 5 Strengths and Limitations

A major strength of this study was the high adherence to CPAP therapy with minimal residual AHI, ensuring robust evaluation of treatment effects. However, this study had some limitations. First, the modest sample size (*n* = 51) precluded the possibility of comprehensive subgroup analyses to explore more potential modifiers of the blood pressure response to CPAP. Second, selection bias may have existed because we exclusively enrolled treatment‐naïve patients who were strongly motivated to avoid antihypertensive medications. Although clinically relevant, this population may not fully represent the broader population with OSA and hypertension. Third, ABPM was conducted during hospitalization to standardize measurements and reduce activity‐related variability, but this may limit applicability to daily‐life conditions. Fourth, the follow‐up period was only 3 months, which may not reflect the long‐term impact of CPAP on blood pressure. Larger studies conducted over longer periods are required to validate these findings.

## 6 Conclusions

CPAP therapy induces clinically modest blood pressure reduction in treatment‐naïve patients with OSA and newly diagnosed hypertension. The treatment effect was most pronounced in patients with elevated baseline blood pressure (24‐h MAP ≥ 96 mmHg), supporting the use of ABPM to characterize baseline blood pressure profiles, which may help identify patients who could derive greater cardiovascular benefit from CPAP therapy. Future studies should integrate blood pressure measures with OSA‐related risk markers (e.g., desaturation index, sympathetic activity, and metabolic profiles) to better predict CPAP treatment response.

## Author Contributions

**Yuanni Jiao:** conceptualization, data curation, formal analysis, investigation, methodology, visualization, writing – original draft. **Hehe Zhang:** data curation, formal analysis, methodology, validation, writing – original draft. **Hao Wu:** validation, writing – review and editing. **Xin Xi:** methodology, writing – review and editing. **Shuang Li:** data curation, writing – review and editing. **Jiang Xie:** funding acquisition, methodology, project administration, resources, supervision, writing – review and editing.

## Funding

The study received financial support from the National Natural Science Foundation of China (Grant No. 82270099). The funding agency had no influence on study design, data collection, or interpretation.

## Ethics Statement

Ethical approval was obtained from the Ethics Committee of Beijing Anzhen Hospital (Approval No. KS2022081). The study complied with the principles outlined in the Declaration of Helsinki. Written informed consent was obtained from all participants prior to their inclusion in the study.

## Conflicts of Interest

The authors declare no conflicts of interest.

## Supporting information


**Table S1:** Polysomnographic measures of the study patients.

**Table S2:** CPAP adherence during follow‐up.

## Data Availability

Data underlying this article can be obtained from the corresponding author upon reasonable request.
