# Supplementary material for: Predicting Significant Blood Pressure Reduction Through Ambulatory Blood Pressure Monitoring in Patients With Obstructive Sleep Apnea Treated With Continuous Positive Airway Pressure
Source: Clin Respir J. 2026 Jan 20;20(1):e70167. doi: 10.1111/crj.70167 (PMC12817275; doi:10.1111/crj.70167)
Supplement: Supplementary file 1 — Table S1: Polysomnographic measures of the study patients. [file CRJ-20-e70167-s002.docx]

**Supplemental table 1. Polysomnographic measures of the study patients.**

| **Variables** | **All patients (n = 51)** | **Patients with baseline 24-h MAP <96 mmHg (n = 20)** | **Patients with baseline 24-h MAP ≥96 mmHg (n = 31)** | ***P* value** |
| --- | --- | --- | --- | --- |
| AHI, (events/h) | 52.7 ± 26.2 | 42.6 ± 27.3 | 59.2 ± 23.7 | 0.032 |
| Oxygen desaturation index, (events/h) | 49.5 ± 25.7 | 37.7 ± 25.9 | 57.1 ± 22.9 | 0.010 |
| CT90, (%) | 17.3 (3.6, 42.1) | 5.3 (2.3, 39.0) | 21.4 (7.6, 43.6) | 0.103 |
| Minimum oxygen saturation, (%) | 71.8 ± 10.7 | 76.9 ± 10.0 | 68.6 ± 10.0 | 0.007 |
| Mean oxygen saturation, (%) | 93.0 (90.0, 94.0) | 93.5 (90.0, 95.0) | 93.0 (89.0, 94.0) | 0.228 |
| Total arousal index, (events/h) | 47.4 ± 18.5 | 40.5 ± 17.4 | 51.9 ± 18.0 | 0.029 |
| Respiratory arousal index, (events/h) | 17.2 (6.9, 31.2) | 10.9 (3.2, 24.2) | 25.6 (8.1, 35.6) | 0.039 |
| N1 sleep percentage, (%) | 11.7 (6.9, 16.8) | 13.2 (7.2, 16.4) | 11.5 (6.2, 18.9) | 0.923 |
| N2 sleep percentage, (%) | 61.8 ± 12.1 | 62.8 ± 11.0 | 61.1 ± 12.9 | 0.607 |
| N3 sleep percentage, (%) | 11.1 ± 7.0 | 12.4 ± 6.9 | 10.2 ± 7.0 | 0.280 |
| Rapid eye movement sleep percentage, (%) | 12.0 ± 6.4 | 10.4 ± 6.3 | 13.1 ± 6.3 | 0.147 |
| Epworth Sleepiness Scale score | 12.1 ± 5.4 | 10.0 ± 4.8 | 13.4 ± 5.4 | 0.023 |
| Insomnia Severity Index | 5.0 (2.0, 8.0) | 5.5 (2.3, 7.8) | 4.0 (2.0, 11.0) | 0.786 |
| Daytime MAP, (mmHg) | 102.8 ± 10.5 | 92.5 ± 6.4 | 109.5 ± 6.5 | < 0.001 |
| Nighttime MAP, (mmHg) | 92.5 ± 11.0 | 82.3 ± 6.8 | 99.1 ± 7.6 | < 0.001 |
| Daytime heart rate, (bpm) | 85.3 ± 10.9 | 82.3 ± 10.6 | 87.3 ± 10.7 | 0.113 |
| Nighttime heart rate, (bpm) | 69.4 ± 10.1 | 67.0 ± 8.8 | 71.0 ± 10.7 | 0.147 |

Results are expressed as mean ± standard deviation or median (interquartile range).

Abbreviations: AHI = Apnea-hypopnea index; CT90 = Cumulative sleep time percentage with oxyhemoglobin saturation < 90%; MAP = Mean arterial pressure.
