# Supplementary material for: Predicting Significant Blood Pressure Reduction Through Ambulatory Blood Pressure Monitoring in Patients With Obstructive Sleep Apnea Treated With Continuous Positive Airway Pressure
Source: Clin Respir J. 2026 Jan 20;20(1):e70167. doi: 10.1111/crj.70167 (PMC12817275; doi:10.1111/crj.70167)
Supplement: Supplementary file 2 — Table S2: CPAP adherence during follow‐up. [file CRJ-20-e70167-s001.docx]

**Supplemental table 2. CPAP adherence during follow-up.**

| **Variables** | **All patients (n = 51)** | **Patients with baseline 24-h MAP <96 mmHg (n = 20)** | **Patients with baseline 24-h MAP ≥96 mmHg (n = 31)** | ***P* value** |
| --- | --- | --- | --- | --- |
| Median CPAP pressure, (cmH_2_O) | 9.0 ± 2.1 | 8.4 ± 2.1 | 9.4 ± 2.1 | 0.108 |
| 95^th^ percentile CPAP pressure, (cmH_2_O) | 11.5 ± 2.3 | 10.8 ± 2.4 | 12.0 ± 2.3 | 0.104 |
| Residual AHI, (events/h) | 1.7 (0.9, 3.0) | 1.4 (0.8, 2.4) | 2.2 (1.0, 3.2) | 0.180 |
| Median daily usage, (h) | 6.1 (5.0, 6.8) | 6.4 (5.9, 7.0) | 6.0 (4.9, 6.8) | 0.153 |
| Mean daily usage, (h) | 5.7 ± 1.5 | 6.1 ± 1.6 | 5.5 ± 1.4 | 0.189 |
| Percentage of days with CPAP usage ≥4 h, (%) | 83.8 (67.9, 95.0) | 88.5 (80.5, 97.6) | 81.9 (65.7, 94.4) | 0.098 |
| CPAP usage in a week, (d) | 6.0 (4.7, 6.5) | 6.1 (5.3, 6.5) | 5.9 (4.5, 6.6) | 0.462 |

Results are expressed as mean ± standard deviation or median (interquartile range).

Abbreviations: AHI = Apnea-hypopnea index; CPAP = Continuous positive airway pressure; MAP = Mean arterial pressure.
